# Structure of Cellulose Isolated from Rapeseed Stalks

**DOI:** 10.3390/polym17081032

**Published:** 2025-04-11

**Authors:** Bogdan-Marian Tofanica, Emanuela Callone, Elena Ungureanu, Ovidiu C. Ungureanu, Valentin I. Popa

**Affiliations:** 1 “Ion Ionescu de la Brad” Iasi University of Life Sciences, 3 Mihail Sadoveanu Alley, 700490 Iasi, Romania; bogdan.tofanica@iuls.ro; 2 “Klaus Müller” Magnetic Resonance Laboratory, Department of Industrial Engineering, University of Trento, Via Sommarive 9, 38123 Trento, Italy; emanuela.callone@unitn.it; 3 Faculty of Medicine, “Vasile Goldis” Western University of Arad, 94 the Boulevard of the Revolution, 310025 Arad, Romania; 4 “Gheorghe Asachi” Technical University of Iasi, 73 Prof. dr. docent Dimitrie Mangeron Alley, 700050 Iasi, Romania; vipopa@tuiasi.ro

**Keywords:** rapeseed stalks, cellulose, α-cellulose, CP/MAS ^13^C NMR, FTIR

## Abstract

Solid state Cross-Polarization/Magic-Angle-Spinning ^13^C CP/MAS Nuclear Magnetic Resonance (NMR) spectra were obtained for cellulose and α-cellulose isolated from rapeseed stalks. This study provides the first characterization of the rapeseed stalk cellulose, revealing that native cellulose occurs as cellulose I allomorph, while α-cellulose exhibits distinct crystalline structures similar to those found in cellulose II. Additionally, Fourier Transform Infrared (FTIR) spectroscopy, Scanning Electron Microscopy (SEM), and Energy-Dispersive X-ray Microanalysis (EDX) were employed to further investigate and unveil the structural properties of cellulose extracted from rapeseed stalks. These complementary techniques offered a more comprehensive understanding of the cellulose morphology, crystallinity, and chemical composition, providing valuable insights into the potential utilization of rapeseed stalks as a renewable biomass resource for various industrial applications.

## 1. Introduction

Cellulose [1], the Earth’s most abundant biopolymer, exhibits a remarkable versatility, underpinning its presence and fundamental role in both natural and manufactured environments. As the primary structural component of plant cell walls, cellulose serves a broad range of applications, from traditional construction materials to advanced biomaterials [2]. Its inherent fibrous nature, evident in materials like cotton and wood pulp, provides the tensile strength essential for textiles, pulp, and paper products. As a macromolecular entity, cellulose can be further processed—dissolving pulp, for instance, acts as precursor for regenerated fibers and cellulose derivatives, expanding its utility in advanced materials [3]. Moreover, the emerging field of cellulosic biofuels highlights the potential of this macromolecule as a renewable energy source.

Recognizing the importance of resource conservation, the well-established practice of recycling cellulose-based materials, especially paper and wood products, highlights its role in supporting circular economy principles [4]. Consequently, ongoing research into optimizing cellulose extraction, modification, and recycling processes, including the exploration and utilization of novel cellulose sources, remains crucial for addressing global sustainability challenges and fostering innovation in material development [3,4,5].

While the traditional cellulose sources, particularly wood pulp, have historically dominated industrial applications since the Industrial Revolution, growing awareness of environmental sustainability has driven significant interest in alternative feedstocks. Notably, before widespread industrialization, annual plants were the primary raw material for cellulose production [6]. Concurrently, the increasing generation of agricultural waste necessitates the development of effective utilization strategies [7].

The conversion of agricultural residues, such as crop stalks and husks, into valuable cellulose-based resources is driven by several critical factors. First, the sheer volume of these residues generated annually poses a significant waste management challenge. Second, the rising demand for sustainable materials calls for a transition away from reliance on conventional, often forest-derived, cellulose sources. Third, these residues represent an abundant and renewable feedstock, making their utilization a key element in fostering a circular bioeconomy. This approach directly addresses the challenging need for development of environmentally responsible industrial practices.

Building on the recognition of agricultural residues as valuable resources, *Brassica napus* L. (rapeseed) emerges as a particularly compelling case. This crop is primarily cultivated for its high-quality oil and protein cake. While the seed is the most valuable harvested component, the stalks are typically used in crop rotation to both enhance soil humus content and facilitate the incorporation, immobilization and transformation of available nitrogen and sulfur into stable organic forms in nutrient-poor soils [8].

Rapeseed stalks, an abundant agricultural byproduct, occur in large quantities at low costs. They contain significant amounts of polysaccharides and lignin, key raw materials for a variety of integrated biorefinery concepts in the emerging bioindustry as well as conventional industrial processes. In rapeseed stalks, holocellulose content ranges between 70–75%, with α-cellulose accounting for 38–42%, lignin for 17–21% and ash for 2–5% [9,10,11,12,13,14]. This compositional profile highlights the potential of rapeseed stalks as a valuable source of raw materials, warranting further investigation into their structural characteristics and potential application in sustainable production.

Given their significant chemical composition and sustainable resource potential, this study aims to isolate and comprehensively characterize cellulose derived from rapeseed stalks. Specifically, Nuclear Magnetic Resonance (NMR) and Fourier-Transform Infrared (FTIR) spectroscopy will be employed to determine the chemical composition and structural attributes of the extracted cellulose. Additionally, the study will encompass an analysis of extractives and inorganic constituents within the rapeseed stalks, thereby providing a holistic understanding of this raw material composition. Scanning Electron Microscopy (SEM) will be used to visualize the morphological features of the isolated cellulose, offering insights into its structural organization.

The elucidation of the native cellulose structure within rapeseed stalks has critical implications for understanding the transformation pathways of these lignocellulosic biomass feedstocks. This knowledge is essential for assessing cellulose behavior both as a fiber constituent and a macromolecule in various materials [15,16,17], as well as for advancing the use of alternative raw materials and biorefinery strategies to produce bio-based products, including materials, fibers, chemicals, and energy [18].

## 2. Materials and Methods

**Figure 1:**
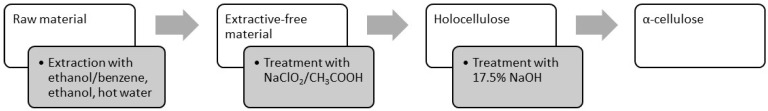
Method for determination of chemical components in rapeseed stalks.

The methods commonly used to study wood and nonwood chemical compositions [10,19,20], based on standardized procedure and generally approved methods from the literature, were used for determination, isolation and purification of chemical components in rapeseed stalks (TAPPI methods, ISO standards) [21]. Figure 1 shows the scheme of the experimental procedure.

Rapeseed stalks, free of leaves and dust, were harvested after seed collection from Vaslui County, Romania. The harvested stalks were air-dried and subsequently stored under laboratory conditions. Prior to testing, the stalks were manually segmented into 3–5 cm lengths. Moisture content determination was conducted in accordance with Technical Association of the Pulp and Paper Industry (TAPPI) standard T258 om-06—Basic Density and Moisture Content of Pulpwood, employing oven drying at 105 ± 3 °C.

For chemical compositional analysis, the stalks were ground and sieved to achieve a uniform particle size, passing through a 0.40 mm screen, as per TAPPI standard T257 cm-02—Sampling and Preparing Wood for Analysis.

The raw material underwent sequential extraction: initially, with an ethanol–benzene mixture (1:2) for 8 h under reflux, followed by ethanol extraction for 4 h under reflux, and finally with boiling distilled water for 1 h, adhering to TAPPI standards T204 cm-07—Solvent Extractives of Wood and Pulp and TAPPI T264 cm-07—Preparation of Wood for Chemical Analysis. The resulting extractives were concentrated using a rotary evaporator, and the solvent was removed, yielding the extractable fraction in solid form. Extractive-free material samples were utilized for the determination of holocellulose content and α-cellulose content within the raw material by a mild delignification step with an acidified solution of sodium chlorite to remove the lignin [22,23], and then with strong hydroxide solutions of NaOH (TAPPI Standard T 203 cm-09—Alpha-, Beta-, and Gamma-Cellulose in Pulp) to obtain undegraded high-molecular-weight cellulose, the “classical” α-cellulose [24,25]. All analytical results are reported on an oven-dry weight basis.

Following the extraction and purification processes, three distinct materials were obtained: extractive-free samples, holocellulose, and α-cellulose. These materials were subjected to analysis to determine their principal chemical composition using NMR and FTIR spectroscopy.

Solid-State ^13^C CP/MAS NMR was used in the sample preparation for structural analysis of extractive-free, holocellulose and α-cellulose [26,27]. Solid-state NMR experiments were carried out on a Bruker Avance 400 WB spectrometer (Bruker Biospin, Billerica, MA, USA), operating at 100.613 MHz for ^13^C. Samples were packed in 4 mm diameter zirconia rotors. Experimental conditions: ^13^C ramp CP-MAS: 2.7 μs for 90° pulse, 5 s for recycle delay, 2 ms of contact time, and 10 kHz of rotating speed. Tetramethylsilane—Si(CH_3_)_4_ (TMS) was used as a primary shift scale reference for ^13^C analyses.

FTIR spectroscopy was performed using a Bruker Vertex 70 spectrometer (Bruker Optics, Billerica, MA, USA) on potassium bromide (KBr) pellets with a 2 cm^−1^ resolution. The concentration of the samples was a constant of 2 mg/200 mg of KBr [28]. Spectral data were analyzed using Spectragryph—Optical Spectroscopy Software. Version 1.2.16.1 (2025), an optical spectroscopy software Spectragryph [29]. Scanning electron microscope (SEM) images were obtained with JSM 5500 Jeol (Jeol LTD, Tokyo, Japan) together with energy-dispersive X-ray spectroscopy (EDX) spectra [30,31].

## 3. Results and Discussions

The chemical composition of rapeseed stalks is characterized by a high holocellulose content, reaching 72.1%, which accounts for about three-quarters of the raw material’s weight. The stems contain approximately 40% α-cellulose and 32% hemicelluloses, along with significant amounts of soluble substances (extractable in cold water, warm water, and 1% NaOH solution) [11]. The chemical composition of rapeseed stems is similar to that of cereal straw, exhibiting a high ash content and pentosans, which are typical for annual plants [7]. Additionally, rapeseed stalks contain a considerable amount of extractable substances, ranging from 5 to 10%, including waxes, fats, and resins, which are soluble in organic solvents such as ethanol–benzene and ethanol.

**Figure 2:**
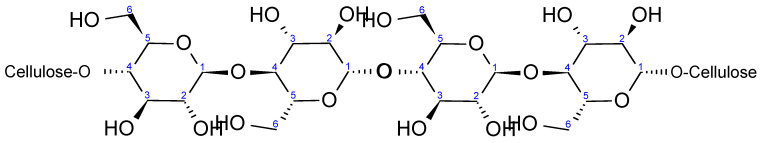
Schematic representation of cellulose I (native cellulose) structure.

Cellulose is the primary constituent of plant tissues, accounting for approximately half of the dry matter in biomass and serving as the fundamental framework of the vegetable kingdom [24]. From Figure 2, it can be seen that cellulose is a homopolymer of β-D-glucopyranose units arranged in a ^4^C_1_ chain conformation, linked together by glycosidic bonds [32], with glucose as the repeating unit [33]. In cellulose, carbon atoms 2, 3 and 6 bear free hydroxyl groups that are available for chemical reactions [34]. However, every carbon is bonded to at least one oxygen, except for C-1, which is connected with two oxygen atoms.

**Figure 3:**
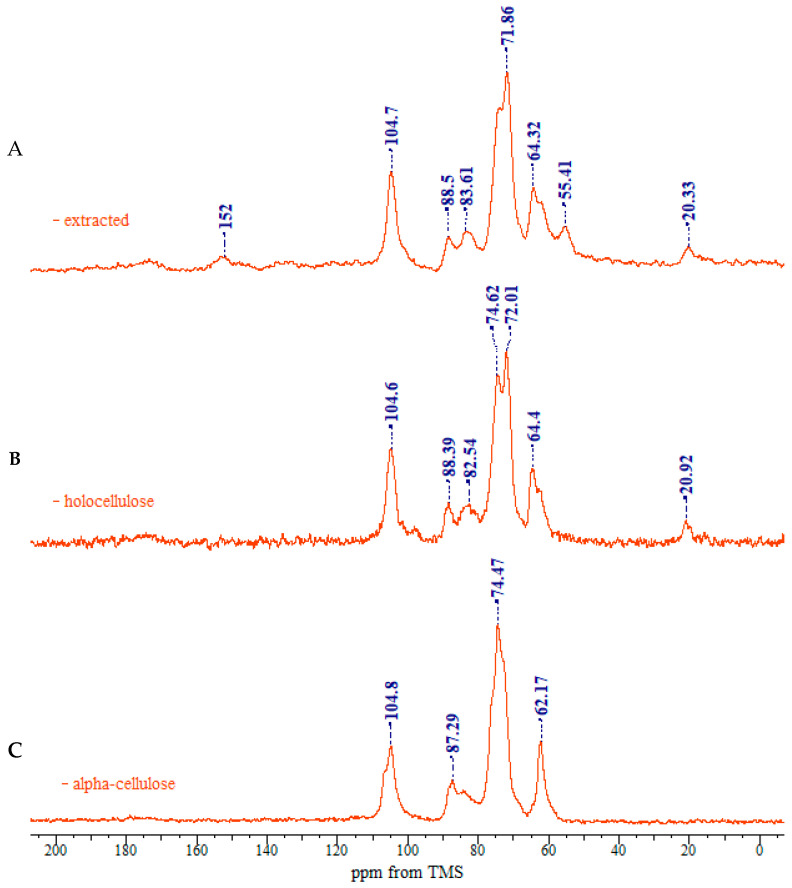
CP/MAS ^13^C NMR spectra of rapeseed stalks: (**A**) extractive-free, (**B**), holocellulose, and (**C**) α-cellulose.

Figure 3 shows the ^13^C CP/MAS NMR spectra of the representative samples. The resonances in the range 50–110 ppm refer to the carbons bonded to oxygen in the cellulose structure. Both chemical shift and line shape help the discrimination of the carbons signals in the anhydroglucose unit; therefore, the description of cellulose polymorphs can be predicted. Broad resonances in the 180–120 ppm range belong to the residual lignin fraction.

**Figure 4:**
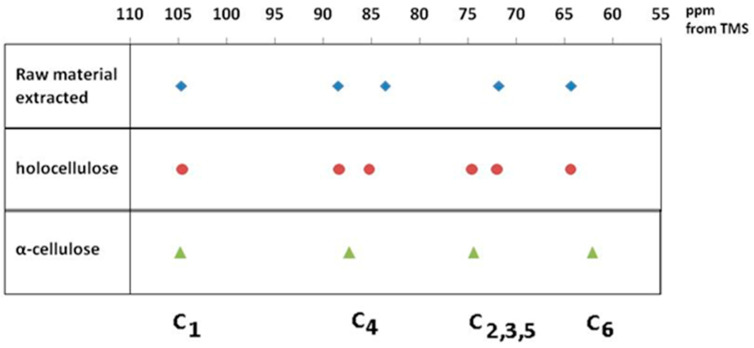
Main peak assignment of cellulose samples.

Assignment of the spectra was made with the aid of previous works by Atalla and Vander Hart and [35,36,37] is given in Table 1 and Figure 4.

In the upfield region of the spectrum, resonances between 60 and 70 ppm are assigned to C-6 of the primary alcohol group. The composite peak between 70 and 81 ppm is attributed to the ring carbons C-2, C-3, and C-5, excluding those involved in the glycosidic linkage. The region between 81 and 93 ppm is associated with C-4, both crystalline and amorphous, while the range between 102 and 108 ppm is associated with the anomeric carbon C-1. The ^13^C NMR spectra of rapeseed stalk extractive-free and holocellulose samples, as shown in Figure 3A,B are quite similar, indicating that the cellulose structure remained relatively unaffected by the treatment for the lignin removal.

Native cellulose is metastable, and it is classified as cellulose I. It features a parallel chain-packing arrangement consisting of two distinct crystalline forms, Iα and Iβ, with their proportion varying depending on the cellulose source. The cellulose I structure exhibits a complex and disordered hydrogen-bonding network. Two distinct intra-molecular hydrogen bonds are present (one between the O3-H and adjacent ring O5′, and another between the O2-H and the neighboring glucose unit O6′). Additionally, one intermolecular hydrogen bond between O6-H and O3′ is observed [38]. Between the molecular layers, van der Walls forces [39] and potential weak hydrogen bonds [40,41] contribute to the structural cohesion.

As shown in Figure 3C, the ^13^C NMR spectrum of the α-cellulose sample is typical of cellulose II, as evidenced by the splitting of the ring carbon C-1 signal [42]. The region between 70 and 81 ppm, corresponding to C-2, C-3 and C-5, differs from that of the extractive-free and holocellulose samples. These variations may be linked to changes in hydrogen-bonding patterns, which allow the cellulose chain to adopt a new molecular conformation. The crystalline structure of cellulose II features an antiparallel chain-packing arrangement with a complex network of hydrogen bonds within and between molecular layers [43]. This extensive hydrogen bonding confers greater stability on cellulose II, compared to cellulose I.

**Figure 5:**
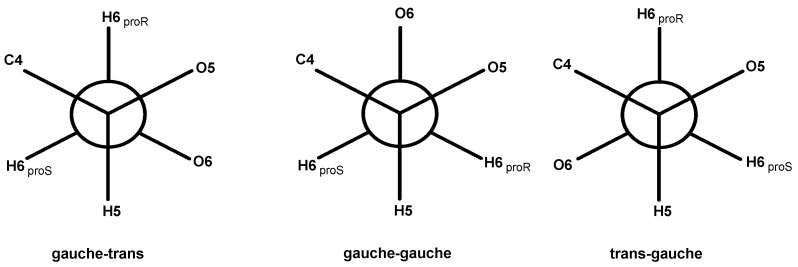
Rotational positions of the hydroxymethyl group: gauche-trans (gt), gauche-gauche (gg) and trans-gauche (tg).

The occurrence of the C-6 resonance as a singlet at 62 ppm indicates a conformational shift [44], specifically from the *tg*-rotamer, characteristic of the glucose unit in cellulose I, to a *gt* conformation for the hydroxymethyl group in cellulose II, as illustrated in Figure 5 [39,40].

The α-cellulose, isolated after treatment with 17.5% NaOH, followed by washing and neutralization, differs from native cellulose, not only in lattice dimensions but also in its degree of crystallinity [45].

The transition from cellulose I to cellulose II polymorphs is further identified by an increase in the relative intensity of the C-6 signal at 62 ppm, which is associated with the amorphous regions of cellulose, and a corresponding decrease in the signal at 64 ppm, associated with the crystalline regions of cellulose I. Moreover, the C-4 resonances belonging to the crystalline region decrease in intensity, while the C-4 related to the amorphous region expands into a broader resonance [46].

The polysaccharides that make up the cell wall of rapeseed stalks primarily consist of hexoses (D-glucose, D-mannose, D-galactose, and L-galactose), pentoses (D-xylose and L-arabinose), 6-deoxy-hexoses (L-rhamnose and L-fucose), and hexuronic acids (D-galacturonic acid and D-glucuronic acid) [47]. Hemicelluloses represent a significant group of plant-derived polysaccharides, ranking second in abundance after cellulose. The monomeric units of hemicelluloses associated with cellulose in the plant cell wall include hexoses, pentoses, and uronic acids, the latter resulting from the oxidation of hexoses.

Rapeseed contains common terrestrial plant polysaccharides that fulfill both structural and physiological functions. The hexose content in whole rapeseed stems ranges from 8.8% to 10.5% (determined by difference). The pentosan yield from whole stems was 23.4%, reaching up to 33.7% in the pith tissue.

**Figure 6:**
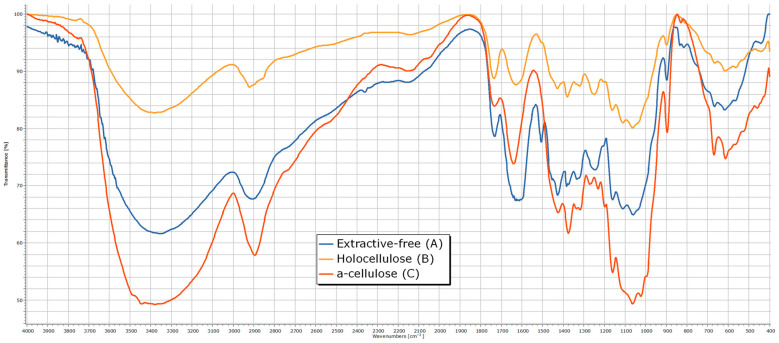
The IR spectrum of rapeseed stalks—extractive-free (A), holocellulose (B) and α-cellulose (C).

The analysis of the IR spectra (Figure 6) reveals distinct absorption bands corresponding to the functional groups of carbohydrates: a broad OH stretching band at 3354 cm⁻^1^, indicative of strong hydrogen bonding, and CH, CH_2_ stretching vibrations at 2906 cm⁻^1^. The presence of lignin in the residue is confirmed by absorption bands at 1732 cm⁻^1^ (carbonyl groups) and at 1632 and 1506 cm⁻^1^, which are characteristic of aromatic structures. The spectral region between 1200 and 1500 cm⁻^1^ contains multiple overlapping absorption bands associated with CH, CH_2_, C=O, and OH functional groups, common to various chemical components. The stretching vibrations of C–O, C–O–C, and C–C bonds within pyranose rings are identified at 1061 and 897 cm⁻^1^.

From the analysis of the IR spectra (Figure 6 and Table 2) for the α-cellulose fraction, the most notable observations include the appearance of absorption maxima corresponding to carboxyl groups at 1230 and 1270 cm⁻^1^, as well as the disappearance of ether bonds between hemicelluloses and cellulose at 1111 cm⁻^1^.

Extractable substances in wood represent the only non-macromolecular components, although they are often associated with polymeric compounds. These substances are present in plant materials in relatively small proportions and can be separated through physical extraction processes. They can be extracted using organic solvents as well as under the action of hot water.

Understanding the content of extractable substances holds significant practical importance, particularly in cellulose manufacturing, as these compounds increase chemical consumption during pulping, inhibit the cooking process, and negatively impact the properties of the obtained cellulose (e.g., color and brightness). A high content of extractable substances leads to reduced cellulose yield in sulfate and natron-AQ pulping processes.

Benzene, ethanol, and their mixtures exhibit maximum extraction capacity and facilitate the removal of waxes, fats, phytosterols, low-molecular-weight carbohydrates, salts, and other compounds.

**Figure 7:**
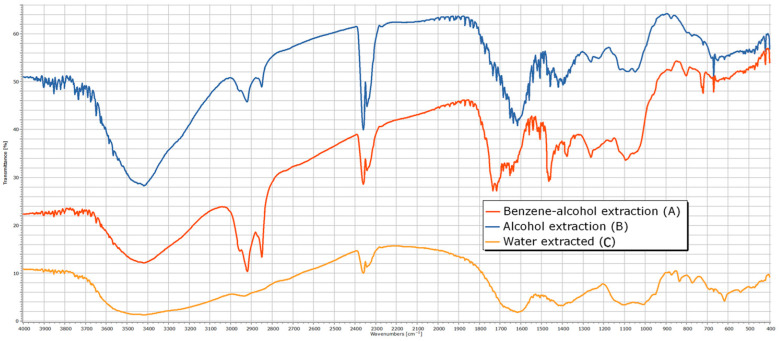
The IR spectrum of extractives from rapeseed stalks—benzene–alcohol extraction (A); alcohol extraction (B); water-extracted (C).

The infrared (IR) spectrum analysis of the extract obtained with an ethanol–benzene mixture can be divided into four characteristic regions (Figure 7), containing absorption bands indicative of specific structural elements:

- Wavelength region 3700–3200 cm⁻^1^—The presence of absorption bands in this region suggests the existence of hydroxyl (-OH) groups characteristic of alcohols (νOH = 3418 cm⁻^1^). The broad appearance of the absorption band indicates hydrogen bonding. Confirmation of the alcohol structure is supported by an additional intense absorption band corresponding to the C-O stretching vibration located in the fingerprint region (νC-O = 1092 cm⁻^1^).
- Wavelength region 3200–2700 cm⁻^1^—Absorption bands in this region correspond to the stretching vibrations of C-H bonds in alkanes (νCH = 2922 and 2853 cm⁻^1^), including asymmetric and symmetric stretching vibrations of methylene (-CH_2_-) groups present in alkanes, such as R(CH_2_)_4_-C, R(CH_2_)_4_-OR, and R-CH_2_-R. The presence of these functional groups is further confirmed by absorption bands corresponding to alkane C-H stretching vibrations, particularly in the νCH = 1377 cm⁻^1^ region of the IR spectrum.
- Wavelength region 1900–1400 cm⁻^1^—This region exhibits intense absorptions primarily attributed to the stretching vibration of the heterogeneous C=O bond (νC=O = 1734 cm⁻^1^), which is characteristic of functional groups such as aldehydes, ketones, carboxylic acids, and esters. Additionally, this region contains bands indicative of C=C double bonds (νC=C = 1647 cm⁻^1^), which may appear less intense but are highly useful in structural assignments for alkenes and aromatic compounds. The absorption band at 1508 cm⁻^1^ is characteristic of benzene ring vibrations.
- Wavelength region 1400–400 cm⁻^1^ (fingerprint region)—This region contains numerous absorption bands that characterize the overall molecular structure, including skeletal vibrations such as deformation, combination, and harmonic vibrations that cannot be assigned to normal vibrational modes. This region is particularly useful for compound identification by comparison with reference IR spectra. Notable absorptions include those associated with C-H stretching vibrations in alkanes, alkenes, and aromatic hydrocarbons (νCH = 1377 cm⁻^1^), as well as C-O stretching vibrations in alcohols, ethers, esters, and carboxylic acids (νC-O = 1263 cm⁻^1^). Additionally, C-H deformation vibrations (both in-plane and out-of-plane) in alkanes, alkenes, and aromatic hydrocarbons appear at δC-H = 725 and 650 cm⁻^1^, respectively. The presence of C-O-C bonds is confirmed by asymmetric (νC-O-C = 1092 cm⁻^1^) and symmetric (νC-O-C = 806 cm⁻^1^) stretching vibrations, which are characteristic of pyranose derivatives.

The extraction of plant material represented by rapeseed stems using ethanol shows both similarities and differences when compared to the extraction with a mixture of ethanol–benzene. The absorption bands in the wavelength regions 3200–2700 cm^−1^ and 3200–2700 cm^−1^ are similar, with both extracts removing alcohol derivatives (with vibration maxima at 3416 and 1080 cm^−1^) and alkanes (2922, 2851, and 1385 cm^−1^) from the plant material. The spectral maxima observed in the characteristic spectrum include a peak at 1603 cm^−1^ (due to bending vibrations of C=O groups in ketones such as C-(C=O)-C=C-OH), a peak at 1267 cm^−1^ (due to bending vibrations of C-O-C groups found in ethers), and a peak at 1049 cm^−1^ (due to bending vibrations of C-O groups typical for alcohols and esters).

The examination of the IR spectrum of the hot water extract can be divided into four representative regions, which contain characteristic absorption bands determined by the presence of structural elements:

- Wavelength region 3700–3200 cm^−1^: The presence of absorption bands in this region suggests the presence of OH groups, indicating the structure of an alcohol (νOH = 3420 cm^−1^). The broad nature of the absorption indicates hydrogen bonding. To confirm the alcohol structure, an additional absorption band due to the stretching vibration of the C-O bond in the fingerprint region (νC-O = 1103 and 1011 cm^−1^) is identified.
- Wavelength region 3200–2700 cm^−1^: The absorption bands in this region are attributed to the stretching vibrations of C-H bonds in alkanes (νCH = 2936 cm^−1^ and a shoulder at 2830 cm^−1^), as well as asymmetric and symmetric stretching vibrations specific to methylene groups in alkanes: R(CH_2_)_4_-C, R(CH_2_)_4_-OR, and R-CH_2_-R. To confirm the presence of these functional groups in the molecule, additional absorption bands due to the stretching vibration of C-H bonds in alkanes located at the characteristic νCH = 1398 cm^−1^ region of the IR spectrum should be identified.
- Wavelength region 1900–1400 cm^−1^: In this region, the spectral maxima characteristic of bending vibrations of C=O groups in ketones of the type C-(C=O)-C=C-OH (δC=O = 1616 cm^−1^) appear, as well as an absorption band at 1506 cm^−1^, which is typical for the benzene ring nucleus.
- Wavelength region 1400–400 cm^−1^ (fingerprint region): This region contains numerous intense absorption bands, but their structural significance is minor.

**Figure 8:**
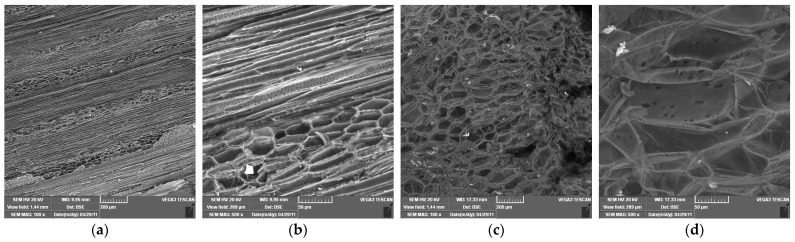
Scanning electron microscopy of rapeseed stalks at different magnifications. (**a**) Tangential section 100×; (**b**) tangential section 500×; (**c**) cross section 100×; (**d**) cross section 500×.

The SEM images provide valuable insight into the microstructural characteristics of rapeseed stalks, which are crucial for understanding their mechanical properties, processing potential, and applications in bio-based materials. For SEM images of rapeseed stalks in the tangential section at 100× and 500× magnifications (Figure 8a,b), it can be observed as follows:

- At 100× magnification: The overall cellular structure of the rapeseed stalk becomes visible, showing distinct fibrous regions along with the parenchyma tissue in the core. The fibers appear elongated and well organized, contributing to the mechanical strength of the stalk. The parenchyma cells, which are more loosely arranged, can be identified in the central region, serving as a storage and transport tissue.
- At 500× magnification: The finer details of the fibrous network are more pronounced. The individual fibers appear more distinct, with their elongated structure and thick cell walls visible. The parenchyma cells, with their thinner walls and more irregular shapes, can be seen forming a matrix around the fibers. The contrast between these two tissue types highlights the hierarchical structure of cells in the rapeseed stalk, with the fibers providing rigidity and the parenchyma contributing to flexibility and metabolic functions.

In the SEM micrographs of a rapeseed stalk core at 100× and 500× magnification (Figure 8c,d), the cross-sectional structure reveals specific anatomical features characteristic of lignocellulosic plant materials:

- At 100× magnification, the overall cellular organization is visible, highlighting the differentiation between the outer protective layers and the inner core. The parenchyma in the central region appears as a porous, loosely packed structure, with thin-walled cells forming an interconnected network. This tissue plays a key role in storage and transport, and its open structure suggests a high potential for moisture retention and enzymatic accessibility.
- At 500× magnification, finer details of the parenchyma become more apparent, showing individual cell walls and the varying sizes of lumen spaces. The parenchyma cells appear somewhat collapsed or deformed in certain areas, likely due to the drying process, but their overall arrangement remains discernible.

The contrast between the outer region and the core of the stalks highlights the heterogeneity of the composition, which influences its mechanical properties and potential applications in bio-based materials.

**Figure 9:**
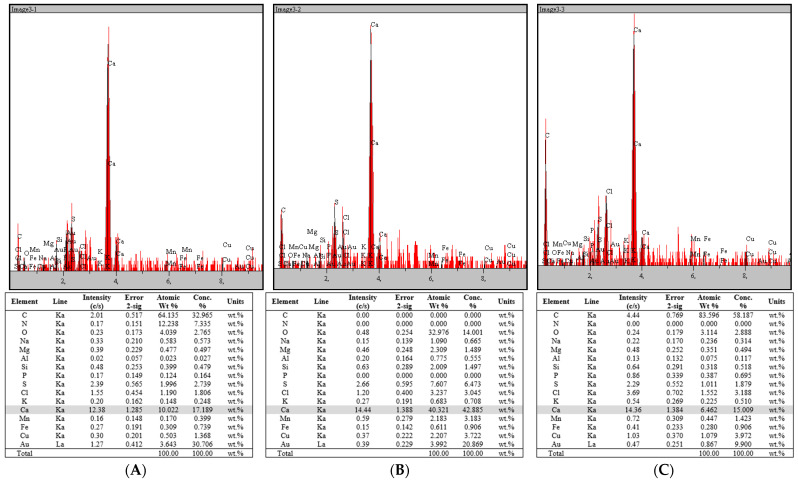
EDX spectra of rapeseed stalks: (**A**) extractive-free, (**B**), holocellulose, and (**C**) α-cellulose.

The mineral composition of rapeseed stalks, totaling approximately 6%, was analyzed using EDX (Figure 9), revealing a combination of soluble salts predominantly consisting of carbonates, sulfates, chlorides, and oxalates, along with an insoluble fraction made up of silicates, phosphates, calcium oxide, magnesium oxide, ferric oxide, manganese oxides, and other compounds. Elemental analysis of the rapeseed stalk samples (Figure 9) indicates that calcium (Ca) is the most abundant metal, with trace amounts of sodium (Na), magnesium (Mg), aluminum (Al), silicon (Si), potassium (K), manganese (Mn), iron (Fe), and copper (Cu) also detected.

## 4. Conclusions

The structural characteristics of rapeseed cellulose are of critical importance for the future utilization of rapeseed stalks as a valuable cellulose source for the production of various chemicals and materials. The main transformation detectable in the crystalline structure following treatment of holocellulose with 17.5% NaOH is the conversion of the crystal lattice from cellulose I to cellulose II. In addition, a decrease in the crystalline region of the C-4 and C-6 visible from the NMR spectra is observed, along with a reduction in degree of crystallinity and conformational change in the hydroxymethyl groups at the C-6 position.

Infrared spectroscopy provided key insights into the structural features of rapeseed stalks, identifying functional groups associated with carbohydrates, lignin, and other extractable substances. Notable spectral features include a broad OH stretching band, characteristic C-H stretching vibrations, and the presence of lignin-related aromatic structures. The IR spectra also facilitated the differentiation of the chemical components within the extractives, offering insights into the molecular composition of the extractable substances, which affect the processing and quality of cellulose extracted from the rapeseed stalks.

The study also highlighted the presence of extractable substances, such as alcohols, alkanes, and waxes, in rapeseed stalks. These extractives can significantly impact the cellulose production process, particularly in pulping, by increasing chemical consumption, inhibiting the cooking process, and reducing both quality and yield of cellulose. Therefore, understanding and managing these substances is crucial for optimizing cellulose extraction from rapeseed stalks.

Scanning Electron Microscopy (SEM) images provided valuable insights into the microstructure of rapeseed stalks, revealing a well-organized fibrous network and the structural arrangement of parenchyma tissue. The fibers were found to be highly organized, contributing to the mechanical strength of the stalk, while the parenchyma tissue played a key role in flexibility and metabolic functions. The distinct structure between the outer protective layers and the inner core highlighted the rapeseed stalk potential for various bio-based applications.

The mineral composition of rapeseed stalks, accounting for approximately 6% of their weight, includes both soluble and insoluble salts, with calcium being the most abundant element. Other trace elements, such as magnesium, sodium, and iron, further contribute to the complex mineral profile of rapeseed stalks. This mineral composition may influence both the chemical properties and the suitability of rapeseed stalks for various industrial applications.

Overall, this study suggests that rapeseed stalks, with their high cellulose and hemicellulose content, along with their unique structural and mineral composition, hold significant promise as a renewable resource for bio-based materials. However, the presence of extractives and minerals must be carefully considered during processing to optimize material yield and quality, particularly in the context of cellulose production.

## Figures and Tables

**Table 1:** Assignment of chemical shifts in the NMR spectrum of rapeseed stalks.

Functional Group	Extractive-Free	Holocellulose	α-Cellulose
Alkyl (0–50 ppm)	20.34 (methyl in H)	20.92 (methyl in H)	n.d.
Methoxyl (50–60 ppm)	55.41 (methoxyl in L)	n.d.	n.d.
O-alkyl (60–110 ppm)	62.05 (C6 in C) 64.33 (C6 in C) 71.86 (C2, C3, C5 in C) 73.85 (C2, C3, C5 in C) 83.61 (C4 amorphous C) 88.50 (C4 crystalline C) 104.66 (C1 in C)	62.95 (C6 in C) 64.40 (C6 in C) 72.01 (C2, C3, C5 in C) 74.62 (C2, C3, C5 in C) 82.54 (C4 amorphous C) 88.39 (C4 crystalline C) 104.64 (C1 in C)	62.18 (C6 in C) - - 74.47 (C2, C3, C5 in C) 84.25 (C4 amorphous C) 87.29 (C4 crystalline C) 104.81 (C1 in C)
Aromatic (110–145 ppm)	134.66 (aromatic in L)	n.d.	n.d.
Phenolic (145–160 ppm)	151.98 (phenolic in L)	n.d.	n.d.
Carbonyl (160–200 ppm)	(173.5) (carbonyl in H)	(174.48) (carbonyl in H)	n.d.

Abbreviation: C—cellulose, H—hemicellulose, L—lignin, n.d.—not detected.

**Table 2:** Main absorption bands in the IR spectrum of rapeseed stalks.

Absorption Bands (cm^−1^)	Vibration Type *	Intensity **	Chemical Bond ***	Functional Group	Extractive-Free	Holocellulose	α-Cellulose
3650–3000	ν (O-H)	s	H-OH; R-CH_2_-OH; (R)_2_CH-OH; (R)_3_C-OH Ar-OH	Water, alcohols	3354	3381	3381
3300; 3100–3000 3000–2800	ν (C-H)	s; m; s	≡C-H; =C-H; -C-H	-	2906	2920	2897
1820–1680	ν (C=O)	vs	R-HC=O; R-CO-OH C=C-CO-O-R Ph-CO-O-R	Aldehydes, carboxylic acids, esters, phenolic esters	1732	1736	1732
1900–1500	ν (C=C)	0-w	-HC=CH-	Alkenes	1632	1630	1641
1525–1470	ν (C=C), aromatics	m-s	Ar-R	Phenols: m-disubstituted, p-disubstituted, 1,3,4-substituted, 1,3,4,5-substituted	1506	-	-
1440–1395	δ (O-H)	w	-CO-OH	Carboxylic acids	1427	1429	1425
1450–1330	δ (O-H)	s-m	-R-OH	Alcohols	1383	1379	1375
1400–1300 1350–1320	δ (C=O) δ (O-H)	s	R-CO-OH R-O-H	Carboxylic acids, alcohols	1335	1321	1319
1300–1100	ν (C-O-C)	s	R-OC-O-C	Esters	1244	1252	-
1320–1211	ν (C-O)	s	-C-OC-OH	Carboxylic acids	-	-	1271, 1230
1300–1100	ν (C-O-C)	s	R-OC-O-C	Esters	1161	1163	1161
1150–1060 1125–1090	ν (C-O-C) ν (C-O)	m-s	-H_2_C-O-CH_2_- R_2_HC-OH	Ethers, alcohols	1111	1111	-
1100–1000	ν (C-O)	s	-C-OH	Alcohols	1061	1063	1063

* ν—valence vibrations, δ—elongation vibrations; ** Intensity: s—strong, m—medium, w—weak, 0—very weak; *** R—organic radical, Ar—aromatic nucleus = benzene.

## Data Availability

All the research data obtained during the study are presented in this article, providing a comprehensive analysis of the structural, chemical, and mineral characteristics of rapeseed stalks and their potential applications.
